# HIV and Sexual Health Services Available to Sexual and Gender Minority Youth Seeking Care at Outpatient Public Mental Health Programs in Two California Counties

**DOI:** 10.1089/heq.2020.0014

**Published:** 2020-09-09

**Authors:** Donald Clermont, Todd Gilmer, Jose Luis Burgos, Emily Berliant, Victoria D. Ojeda

**Affiliations:** ^1^ Department of Family Medicine and Public Health and University of California, San Diego School of Medicine, La Jolla, California, USA.; ^2^ Department of Medicine, University of California, San Diego School of Medicine, La Jolla, California, USA.

**Keywords:** sexual and gender minority, public mental health, HIV, youth, young adult

## Abstract

**Purpose:** Sexual and gender minority youth (SGMY, ages 16–24 years) face disparities in sexually transmitted infections (STIs) and HIV, in part, due to exposure to settings and behaviors that may harm youth's physical and mental health. This study examines the scope of sexual health and HIV services available to youth living with serious mental illness (SMI), including SGMY, seeking care at publicly funded outpatient mental health programs.

**Methods:** Between 2018 and 2019, we surveyed 183 managers of mental health programs serving youth living with SMI of ages 16–24 years, including SGMY, in San Diego and Los Angeles counties. Participants reported on programs' target populations, sexual health/HIV service provision, and the use of peer providers. Descriptive statistics and Pearson chi-square tests were used to describe sexual health/HIV services and identify programmatic characteristics associated with providing these services.

**Results:** Overall, 46% of all programs surveyed provided sexual health/HIV services. Of these, 62% provided HIV education, 81% provided sexual/reproductive health education, and 69% provided sexual/reproductive health education tailored for lesbian, gay, bisexual, queer, intersex (LGBQI) youth. Peers often provided these services. Chi-squared tests showed that programs employing peer specialists (*p*=0.009) and targeting LGBQI youth (*p*=0.045) were significantly more likely to provide sexual health/HIV services.

**Conclusion:** The use of peer providers may reduce stigma around sexual/HIV service utilization and promote SGMY's trust. Publicly funded outpatient mental health programs serving youth and especially those actively engaging SGMY may consider also offering onsite HIV, STI, and sexual health services, creating a one-stop-shop approach.

## Introduction

Sexual and gender minority youth (SGMY; e.g., lesbian, gay, bisexual, transgender, queer, intersex, of ages 16–24 years) experience overlapping social identities (e.g., gender, sexual orientation, race/ethnicity, and immigrant status) due to the diverse social groups to which they belong.^1–4^ However, exclusion, stigmatization, and discrimination of these intersecting identities may adversely impact SGMY's mental and physical health outcomes.^1,5^ Specifically, SGMY are at high risk of experiencing mental health disparities while also being at disproportionate risk for sexually transmitted infections (STIs) and HIV.^6–9^ This study describes the availability of sexual health, STI, and HIV services by publicly funded mental health programs that serve SGMY in Southern California, a highly diverse region.

SGMY are significantly more likely to experience conditions that can elevate the likelihood of mental and physical health disparities. Stigma and discrimination by family, peers, and community members due to youth's gender and sexual orientation contribute to adverse mental health outcomes among SGMY,^10–13^ including higher rates of suicidality, depression, and anxiety disorders.^12,14–16^ SGMY experience higher rates of STIs and HIV than heterosexual/cisgender youth,^6–9^ in part because of engagement in risky sexual behaviors (e.g., infrequent condom use, multiple sexual partners, and trading sex) and higher rates of alcohol and illicit drug use—potentially as coping mechanism, which may subsequently contribute to unsafe sexual practices.^17–24^ Importantly, systematic discrimination, such as bullying and physical and sexual abuse, including forced sex or sexual dating violence, has resulted in the overrepresentation of SGMY among homeless or housing insecure youth.^6,25–32^ Notably, African American and Latino SGMY are disproportionately represented among those seeking homelessness services.^26,33^ The numerous adverse social conditions encountered by SGMY can exacerbate their health burden and underscore their need for comprehensive services that can safeguard their mental and physical health.

SGMY experience numerous barriers to receiving needed physical and mental health services (HSs). The Gelberg–Andersen Behavioral Model of Health Services Utilization for Vulnerable Populations can elucidate the factors underlying HS use disparities among SGMY.^34,35^ The Gelberg–Andersen model has been used to study HS utilization among vulnerable communities such as adults experiencing homelessness or serious mental illness (SMI).^36–38^ The model notes that predisposing (e.g., demographics), enabling (e.g., individual and community resources), and need characteristics (e.g., individual perceived need and professionally evaluated need) interact to influence health care utilization. Enabling factors play an important role in youth's health care access as they may experience disruptions in health care as they exit child/adolescent HSs and enter into adult HSs. The model further identifies areas of vulnerability relevant to SGMY such as sexual orientation, victimization, family resources, and youth's access to public benefits.^39,40^ Access to care barriers for youth includes confusion about navigating the insurance system or the cost of coverage, thus financial barriers may be important factors limiting youth's HS utilization.^41,42^ Characteristics of the health system may also impact SGMY's engagement and range from limited provider training regarding SGM health to stigmatization of SGM individuals and assumptions of patients' heteronormativity by clinicians.^42^ Consequently, SGMY may mistrust their primary care provider and fear revealing their sexual orientation, resulting in less disclosure about their sexual health needs, HIV risk behaviors, or other factors that elevate SGMY's vulnerability to poor health outcomes. Collectively, this array of individual, family, community, and health system-level factors may contribute to SGMY's unmet needs across physical and social domains that influence health (e.g., conflict management/reduction within families).^42–45^

California has made important progress in the provision of youth-focused mental HSs, facilitated, in part, by the 2004 Mental Health Services Act.^46,47^ For example, in 2017, 30.8% of California's community mental health centers offered treatment programs or groups designed exclusively for youth and SGMY (19.7%) versus 17.8% and 15.7%, respectively, of community mental health centers throughout the United States.^48^ HIV services that are offered by mental health programs enhance care and reduce barriers to accessing needed sexual HSs.^49^ Moreover, integrating sexual health, including HIV services, into mental health care may improve both mental and sexual health outcomes among SGMY by reducing social, administrative, logistical, and financial barriers to care.^49,50^ Yet, we continue to lack a nuanced understanding of the role that mental health programs play in providing sexual health, STI, and HIV services to SGMY who seek mental health care.

Peer-led services have the potential to support SGMY's access to and use of sexual and mental HSs. HIV programs have consistently engaged peers to provide sex education community outreach, and HIV testing.^51–56^ SGMY consider peer support (PS) and guidance to be critical aspects of sexual HSs.^56–59^ and PS may improve health and social outcomes by raising self-efficacy and self-confidence.^60,61^ In addition, those receiving PS exhibit a higher likelihood of returning for annual visits, reductions in risky sexual behavior, and increased self-reported empowerment.^59–61^ PS is increasingly used within mental health settings as they are considered to provide culturally and developmentally appropriate support systems that build on mutuality, empathy, and trust.^62–64^ In 2017, 32.0% of California mental health treatment facilities offered peer services versus 24.6% of US sites.^48^ Less is known about the role of PS in delivering sexual health, STI, and HIV services within mental HS settings serving SGMY.

It is under this backdrop of an evolving mental health and HSs delivery landscape for SGMY that we undertook this study. Using the Gelberg–Andersen Model of Behavioral Health Services Utilization,^34,35^ we focus on enabling factors—that is, we describe the extent to which sexual health and HIV services are available to SGMY within publicly funded mental health programs that serve young adults of ages 16–24 years in California's two largest counties: Los Angeles (2019 population: ∼10.3 million) and San Diego counties (∼3.4 million).^65^ We also describe the role of peers in delivering these services.

## Methods

### Participants

The Los Angeles County Department of Mental Health and San Diego County Department of Behavioral Health Services provided the researchers with lists of programs that served youth of ages 16–24 years who met mental health eligibility criteria (per each program's assessment) and who received outpatient mental health from July 1, 2015, through June 30, 2018. Each program was contacted by phone to describe the study and identify the appropriate respondent. We requested that questionnaire respondents hold a leadership position and be familiar with the program, including the array of services provided and the use of peer specialists. Respondents were primarily program managers and no incentives for participation were provided.

This study was approved by the Human Subjects Research Protections Program at the University of California, San Diego, Los Angeles Department of Mental Health, and San Diego County Department of Behavioral Health Services in accordance with the Privacy Rule of the Health Insurance Portability and Accountability Act of 1996. No client-level data were analyzed for this study.

### Data collection and questionnaires

We implemented computer-assisted self-administered surveys using Qualtrics,™ a Health Insurance Portability and Accountability Act of 1996 (HIPAA)-compliant cloud-based survey software (Provo, UT) to collect program-level data. The survey was conducted from August 1, 2018, to February 30, 2019. Electronic informed consent was provided by participants before responding to the questionnaires. Individuals received personalized survey links and nonresponders or persons with incomplete surveys received weekly e-mailed reminders. Respondents were offered the option of completing an interviewer-administered survey on a day/time of their choosing.

Respondents reported on program characteristics including (1) the provision of HIV and sexual HSs (described hereunder), (2) SGMY communities targeted by the program for outreach and engagement, and (3) whether the program employed peer specialists. Specifically, participants reported on whether the program provided the following services: HIV education, HIV testing, HIV pre-exposure prophylaxis (PrEP), HIV postexposure prophylaxis, or HIV medications. Participants reported on whether the program provided sexual/reproductive health education, sexual/reproductive health education for SGMY, testing for STIs, treatment for STIs, or condoms. Respondents also indicated which staff member was responsible for delivering the aforementioned services, which were reported on individually.

### Statistical methods

We describe program characteristics including the SGMY subgroups targeted for service delivery and the employment of peer specialists, the provision of specific HIV, STI and sexual HSs, and the use of peer specialists to provide these services. We use Pearson chi-square tests to assess the relationship between program characteristics and the provision of HIV and sexual HSs.

## Results

Table 1 gives the characteristics of 183 programs that provide outpatient mental HSs to youth with SMI in Los Angeles and San Diego counties. We requested surveys from 260 programs, resulting in a response rate of 70%. Overall, 46% of outpatient programs serving youth in both counties provide HIV or sexual HSs. Forty-two percent of programs reported having a peer specialist on staff and these programs were more likely to provide HIV or sexual HSs (*p*=0.009).

**Table 1.** Availability of Peer Specialists on Staff and Youth Communities Targeted for Service Delivery by Outpatient Mental Health Programs in Los Angeles and San Diego Counties Stratified by Sexual Health, Sexually Transmitted Infection, and HIV Services Availability

	All programs		Programs targeting a specific youth community that provide HIV, STI, or sexual health services		p^a^
	N	%	N	%	
Overall	183		85	46	
Youth communities targeted by program for outreach and engagement					
LGBQI youth	32	17	20	63	0.045
Transgender youth	26	14	16	62	0.096
MSM youth (i.e., male youth who have sex with men, whether or not they identify as gay or bisexual)	12	7	7	58	0.393
Youth who are homeless or at risk of becoming homeless	44	24	29	66	0.003
Youth who are victims of sex trafficking	24	13	17	71	0.010
Youth who are victims of sexual abuse/violence	35	19	21	60	0.074
Youth with substance use disorders	33	18	19	58	0.157
Youth with dual diagnoses (i.e., mental illness and substance use disorder)	48	26	29	60	0.024
Youth in or exiting the foster care system	37	20	23	62	0.032
Youth involved in the criminal justice system	39	21	22	56	0.160
Parenting or pregnant youth	21	11	15	71	0.015
Peer specialist on staff	76	42	44	58	0.009

Youth are defined as ages 16–24 years.

^a^ For difference in providing HIV or sexual health services among programs targeting a specific youth community.

LGBQI, lesbian, gay, bisexual, queer, intersex; STI, sexually transmitted infection.

Programs targeted multiple SGMY communities including lesbian, gay, bisexual, queer, intersex (LGBQI) youth (17%), transgender youth (14%), youth who are homeless or at risk of becoming homeless (24%), youth who are victims of sex trafficking (13%), youth who are victims of sexual abuse or violence (19%), youth with substance use disorders (18%), youth with dual diagnosis (26%), youth in or exiting the foster care system (20%), youth involved in the criminal justice system (20%), and pregnant or parenting youth (11%).

Programs that were more likely to provide HIV or sexual HSs were more likely to specifically target LGBQI youth, youth who are homeless or at risk of becoming homeless, youth who are victims of sex trafficking, youth with dual diagnosis, youth in or exiting the foster care system, and pregnant or parenting youth (*p*<0.05 each).

Table 2 gives the types of services available among programs that provide HIV and sexual HSs to youth. Among programs providing services, 64% provide at least one of the HIV services queried about and 96% provide at least one type of sexual HS. The most common services were HIV education (62%), sexual/reproductive health education (81%), sexual/reproductive health education activities tailored for SGMY (69%), and condom distribution (45%). Fewer than 10% of programs provide HIV testing, HIV PrEP, HIV medication refills, or testing or treatment for STIs.

**Table 2.** Types of HIV, Sexually Transmitted Infection, and Sexual Health Services Available to Youth in Outpatient Mental Health Programs in Los Angeles and San Diego Counties, Among Programs That Provide at Least One Type of HIV/Sexual Health Service

	Programs providing HIV/sexual health services		Programs with peer specialists who provide HIV/sexual health services		Programs with peer specialists where peers provide HIV/sexual health services	
	N	%	N	%	N	%
Overall	85		44	52		
Provides HIV services	55	64				
HIV education	53	62	27	51	11	41
HIV testing	5	6	3	60	2	67
HIV PrEP	5	6	5	100	1	20
HIV PEP	3	4	3	100	1	33
HIV medications (e.g., refills)	6	7	4	67	0	0
Provides STI and sexual health services	81	95				
Sexual/reproductive health education	69	81	34	49	15	44
Sexual/reproductive health education for SGMY	59	69	32	54	14	44
STI testing	5	6	4	80	1	25
STI treatment	4	5	4	100	0	0
Condom distribution	38	45	26	69	13	50

Youth are defined as ages 16–24.

PEP, postexposure prophylaxis; PrEP, pre-exposure prophylaxis; SGMY, sexual and gender minority youth.

Peers often provided HIV and sexual HSs (Table 2). Overall, 52% of programs providing HIV and sexual HSs had a peer specialist on staff. Among these programs, >40% involve their peer specialists in the provision of HIV education, HIV testing, sexual/reproductive health education, and condom distribution.

## Discussion

This study aimed to address gaps in the research literature describing sexual health, STI, and HIV services offered to SGMY living with mental health challenges and served by publicly funded outpatient mental health programs. We surveyed a large number of programs in San Diego and Los Angeles counties, which are large and racially/ethnically diverse communities in the state. Research studies such as this one are critically needed to help reduce disparities in mental health status as it overlaps with vulnerabilities experienced by subgroups of SGMY. Overall, we found that programs that included peer providers within their staffing models were more likely to provide any sexual health and HIV services. Our study illustrates the challenges that youth living with mental illness may face in accessing sexual health and HIV services through mental health outpatient programs and underscores potential areas of expansion when addressing the integration of mental health and sexual HSs for youth and SGMY living with SMI.

Prior literature asserts that less has been done to identify and meet the needs of SGMY in health care settings.^66,67^ However, youth and SGMY can benefit from accessing tailored services particularly because of the layered experiences of stigma and discrimination that contribute to diverse and complex physical and mental health and social service needs.^1–4^ New research in this area will be required to understand the impact of youth-focused services on clients' outcomes.^68,69^ In 2017, 14.8% of U.S. community mental health centers offered treatment programs of groups specifically dedicated to SGMY clients.^48^ A 2015 survey of publicly funded mental health programs that serve youth throughout California found that SGMY clients constituted an important target population (LGBTQI: 49%), suggesting that Southern California programs may have benefited from California's Mental Health Services Act to create new services for SGMY subgroups.^47^

Our analyses demonstrated that inclusion of peer providers in the program was associated with greater delivery of sexual health and HIV services. A program's use of peer providers may be a marker of organizational preferences for innovation or a funding structure that also allows the program to provide sexual HSs. Peers providers have been demonstrated to increase clients' likelihood of returning for annual visits and reducing risky sexual behavior among SGMY.^59,61^ Thus, mental health programs that serve SGMY may consider engaging SGMY peer providers within their service teams.

### Limitations

Our study relied on survey data and may be impacted by recall bias or respondents' familiarity with clinic operations and staffing responsibilities. In addition, responses may have been accurate as of the time the survey was completed, though conditions may have changed since data collection was terminated. Nevertheless, the large sample of participating programs provides a snapshot of services available to SGMY.

## Conclusion

Given SGMY's life stage, mental health programs that target youth and SGMY communities should consider ensuring that sexual HSs are included within their service array to address health disparities experienced by youth living with mental health challenges and SGMY. A one-stop-shop approach may be effective in addressing multiple challenges to SGMY's service utilization as identified by the Gelberg–Andersen Behavioral Model of Health Services Utilization for Vulnerable Populations.^39,40^ Moreover, an integrated system of care may reduce logistical, systemic, trust, and economic barriers to sexual health and HIV service needs that contribute to disparities in these areas among vulnerable youth and SGMY. Adopting peer providers may aid in the implementation of these services by reducing stigma around service utilization and promoting trust among clients and potential clients. Additional research may better elucidate the barriers to the provision of HIV and sexual HSs and the use of peers within programs that serve SGMY clients.

Future studies may consider including other California counties and states to better assess whether the delivery of sexual health and HIV services is prevalent as well as barriers and facilitators to including these services within mental health programming. In addition, cost-effectiveness studies can demonstrate the impact of these services on SGMY clients' mental and physical health outcomes and racial/ethnic disparities in HIV and STI rates.

## Abbreviations Used

HS: health service
LGBQI: lesbian, gay, bisexual, queer, intersex
PEP: postexposure prophylaxis
PrEP: pre-exposure prophylaxis
PS: peer support
SGM: sexual and gender minority
SGMY: sexual and gender minority youth
SMI: serious mental illness
STI: sexually transmitted infection
